# Rationale and methods of a randomised cross-over cluster trial to assess the effectiveness of MOVI-KIDS on preventing obesity in pre-schoolers

**DOI:** 10.1186/s12889-015-1512-0

**Published:** 2015-02-22

**Authors:** Vicente Martínez-Vizcaino, Jorge Mota, Montserrat Solera-Martínez, Blanca Notario-Pacheco, Natalia Arias-Palencia, Jorge Cañete García-Prieto, Alberto González-García, Celia Álvarez-Bueno, Mairena Sánchez-López, on behalf of the MOVI-KIDS group

**Affiliations:** Health and Social Research Centre, Universidad de Castilla-La Mancha, Edificio Melchor Cano, C/ Teresa Jornet s/n 16071, Cuenca, Spain; Facultad de Ciencias de la Salud, Universidad Autónoma de Chile, Santiago de Chile, Chile; Research Centre in Physical Activity, Health and Leisure, Faculty of Sport, University of Porto, Porto, Portugal; Facultad of Education, Universidad of Castilla-La Mancha, Ciudad Real, Spain

**Keywords:** MOVI-KIDS, Physical Activity, Adiposity, Fitness, Children, Obesity, Cardiovascular Disease, Adiposity Rebound

## Abstract

**Background:**

Childhood obesity has become an alarming worldwide increasing public health problem. The earlier adiposity rebound occurs, the greater the risk of becoming obese during puberty and adolescence. It has been speculated about the potential influence of vigorous physical activity on modifying the age of onset of adiposity rebound. Moreover, studies aimed to evaluate the effectiveness of physical activity interventions programs on reducing adiposity and other cardiovascular risk factors in children younger than 6 years are scarce. This paper describes the rationale and methods of a study aimed to test the effectiveness of a two-years multidimensional pre-school intervention on preventing obesity and improving physical fitness during the adiposity rebound period.

**Methods/Design:**

Twenty-one schools from the provinces of Cuenca and Ciudad Real, Spain, were randomised to an intervention and a control arm. In the first academic year, children in third grade of pre-school and first grade of primary school in the intervention group received the physical activity intervention (MOVI-KIDS). After an academic year schools were crossed over to the alternative arm. According to the socio-ecological model, the intervention included children, their parents and teachers, and the school environment where MOVI-KIDS was conducted. MOVI-KIDS consisted of: i) three-h/week sessions of recreational non-competitive physical activity in after-school time; ii) educational materials to parents and teachers about physical activity benefits and sedentary lifestyle risks; and iii) modifications in the playground to promote physical activity during recess. Baseline and post-intervention outcomes are going to be measured in both arms three times, at the beginning and at the end of first academic year, and at the end of the second academic year. Primary outcomes included body mass index, waist circumference, triceps skinfold thickness, percentage of both body fat and fat-free mass, and blood pressure. Secondary end points were physical activity, fitness, and carotid intima-media thickness.

**Discussion:**

This paper reports the design of a randomised cross-over cluster trial aimed at assessing the effectiveness of the multidimensional physical activity intervention (MOVI-KIDS) during two years in pre-school children.

**Trial registration:**

Clinical trials.gov: NCT01971840. (Date of registration: Initial Release: 10/07/2013; Record Verification: 23/10/2013).

## Background

Childhood obesity is one of the most important public health problems worldwide that in Spain is growing at alarming rate. The prevalence of overweight, including obesity, in the province of Cuenca in 2010 was 35.4% [1]. Similar prevalence figures have been reported in other areas of Spain and other Mediterranean countries [2,3]. The trend to increase the overweight prevalence has also been accompanied by high rates of underweight in children from Spain and other European countries [4].

Obese children are at increased risk to becoming obese in adulthood [5] and to suffer premature cardiovascular events [6]. The risk of obesity in children increases as earlier adiposity rebound occurs [7]. Moreover, child obesity has been related with hypertension, dyslipidemia, platelet aggregation, hyperinsulinemia, osteoarticular problems and carotid intima-media thickness [8,9], all of them predictors of cardiovascular events in adulthood [10]. Conversely to physical fitness, obesity is negatively related to academic achievement, self-esteem, and health-related quality of life [11,12].

Obesity is a complex disorder determined by both genetic and environmental factors, but also by their interaction. The obese child is the phenotypic expression as a result of the interaction between polygenic inheritance with food intake and physical activity (PA) habits, although also social, psychological and environmental factors have been related to the development of obesity during childhood [13]. The relative importance of both energy expenditure and energy intake is a controversial issue in Spain and other countries [14]. In Spanish children, however, some characteristics of the obesity epidemic should be taken into account when we are examining which are the main drivers of this epidemic: i) data from the AVENA study support an association between television watching, videogames, and excess of body fat [15]; and ii) it has been recently described in the adolescents from European HELENA sample that the more physically active and leaner adolescents have higher energy intake than less active adolescents with larger amounts of fat mass [16].

Vigorous PA may have an important role in the prevention of excess of weight in children and overall cardiovascular diseases [17,18]. Furthermore it has been suggested that PA at early ages could increase bone and muscular tissues [19], and could have cardio-metabolic benefits throughout lifespan [20].

Many observations have suggested that the earlier age at adiposity rebound is known to be a risk factor of later obesity because it is associated with a particular body mass index (BMI) trajectory [21]. The age of adiposity rebound depends on genetic factors and environmental and nutritional conditions [22]. Time spent in vigorous PA at early ages might represent a practical strategy to delay the onset of adiposity rebound.

Several studies have assessed the risk factors of obesity and the effectiveness of the interventions to prevent them [15,16,20,23]. The latest Cochrane revision that evaluated the interventions for preventing obesity in children concluded that there is enough evidence of the effectiveness of PA intervention in children from 6 to 12 years old to prevent obesity [24]. Also, this review indicates that there is a lack of evidence in studies to evaluate the effectiveness of these interventions in children younger than 6 years. No study describes in detail the intervention or the cost-effectiveness analysis and none informs about its negative consequences and potential damage. Only one study analysed a health lifestyle promotional program in children of this age, with successful effectiveness in reducing fat mass and improving fitness [23].

Our group has conducted so far two interventions based on after-school programs of recreational PA to control obesity and other cardiovascular risks in primary schools (8 to 11 years old) in Cuenca, Spain. The first edition, called MOVI program, showed a moderate effect in reducing adiposity and improving lipid profile, but did not significantly improve overall cardiometabolic risk, mainly because it did not reduce blood insulin levels [20,25]. A second edition (MOVI-2) increased duration and intensity of the sessions and was more focused on increased muscle strength [26]. It shows a decrease in body fat and fasting insulin, and as a consequence in global cardiometabolic risk levels [27].

This paper reports the rationale and methods of a trial to assess the effectiveness of a multidimensional PA (MOVI-KIDS) promotion intervention in schoolchildren for preventing obesity, improving fitness, and reducing cardiovascular risk during the adiposity rebound period in children from 4-to-7 years of Castilla-La Mancha (Spain). Secondary objectives were to evaluate the effectiveness of this program on increasing the children’s level of PA, their physical fitness and the reduction of carotid intima-media thickness. In addition, a nested qualitative study had as objectives to identify barriers and facilitators to PA in children in the school environment.

This project was coordinated with another project that share the same population-based sample and that was aimed to assess the effectiveness of the PA intervention to prevent obesity and improve academic performance in children with and without Attention Deficit Hyperactivity Disorder (ADHD) risk (ClinicalTrials.gov Identifier NCT01971827).

## Methods/Design

### Study design and participants

**Figure 1:**
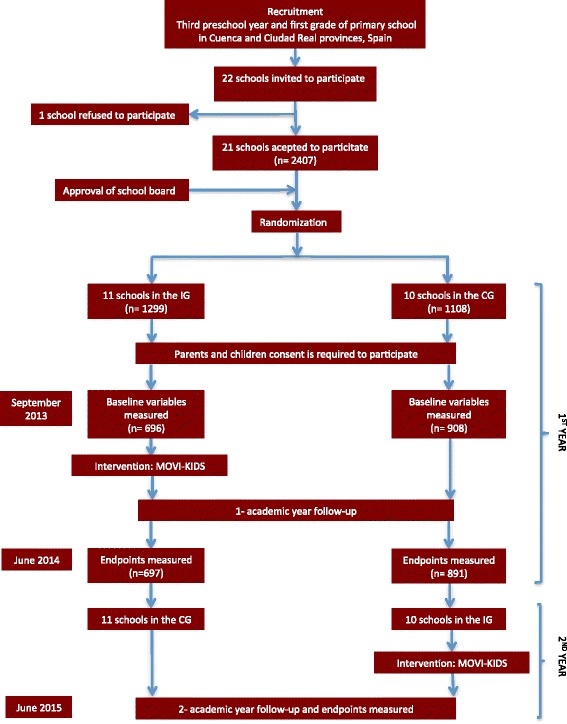
**Flow chart of trial participants.** CG, control group; IG, intervention group.

This was a cross-over randomised cluster trial that involved 21 schools located in as many municipalities in the province of Cuenca and Ciudad Real, Castilla-La Mancha region (Spain). All were public schools except for two private schools, one of each capital province. In municipalities with more than one school, only one was selected for the study. Of the 22 invited schools, only one school refused to participate in the study, arguing that teachers did not accept to participate due to excessive administrative overhead that the study could mean for them. After the approval of School Councils, the schools were randomly allocated by using the statistical package StatsDirect to either the intervention group (IG) or the control group (CG); for this goal the whole sample of schools was divided in three subgroups of randomisation as follows: i) nine public schools from Cuenca; ii) ten public schools from Ciudad Real; and iii) two private schools in the capital of the provinces (Figure 1). In each school, all children belonging to third year of pre-school and first grade of primary school (4-to-7 years old) were invited to participate.

#### Inclusion criteria

To participate in the study the schools had to have at least two full classrooms of third year of pre-school and other two of the first grade of primary school. The approval of Boards of Governors was necessary in order to participate in the intervention and in the measurements at the beginning and the end of the academic year. For all children informed consent from parents or legal representative was a compulsory requirement to participate. The parents were invited to collaborate fulfilling the questionnaires regarding family leisure habits, sleeping, eating, and getting around town.

#### Exclusion criteria

Participants who had severe Spanish language learning difficulties were excluded. Also were excluded participants with serious physical or mental disorders identified by parents or teachers which would impede participation in the program’s activities, and also those with diagnoses of chronic disorders such as heart diseases, diabetes or asthma that in opinion of their paediatricians prevents the participation in the program activities.

### Ethical and legal aspects

The project was supported by the Department of Education and Science of the Junta de Communities of Castilla-La Mancha (Spain), who sent a letter to each school to inform them of the study. Investigators then visited each school to provide information on the study objectives and methods, and to obtain the consent of the head teacher and the school board. This was followed by classroom-by-classroom briefings, in which pupils were asked to collaborate. The MOVI-KIDS study was also presented to physical education teachers. A letter was sent to parents inviting them to a meeting in order to explain the study objectives and procedures. Subsequently parents were asked to sign informed consent approving the participation of their child in the study, with the recommendation to consult the child and to take their views into account.

Data of the child examinations were sent to parents by a letter after each measurement process, and when any anomalous values were detected, the team doctors gave to parents the appropriate recommendations.

The Clinical Research Ethics Committee of the ‘Virgen de la Luz’ Hospital in Cuenca approved the study protocol. An insurance policy was signed to cover any potential risks associated with the intervention.

### Intervention

The design of the MOVI-KIDS is based on the social ecology model [28], a theoretical model of behaviour change. From this paradigm the behaviour is the interaction between the physical and social environment. The MOVI-KIDS program is a multidimensional intervention aimed to influence on individuals (children, families, and teachers) and the environment (including some changes in the physical structure of the schoolyard).

The intervention has been applied in the IG during two full academic years, and was implemented at three levels:

i) Children participated in an extracurricular, adapted to the schoolchildren levels of motor competences (4-to-6 years old), play-based and non-competitive PA program (MOVI-KIDS). The aim of MOVI-KIDS was to increase the weekly PA time through three 60 min sessions/week in the school facilities. The program included basic sports games, playground games, dance and other activities focused on to develop motor skills. At the end of the first year, approximately 90-sessions were performed in each school.
ii) Parents and teachers included in the IG were involved in actions to promote active lifestyles activities in children. These activities included the following: a) use of reinforcement tools that had demonstrated their utility on improving parents’ and teachers’ compliance and involvement in the program (i.e. a refrigerator magnet with recommendations for PA for children); b) answer a satisfaction with the program questionnaire; and c) access to the blog (http://movi3kids.blogspot.com.es/) where parents could observe their children’s progress, read news reinforcing healthy lifestyles, and where parents could asking questions or making complaints to the research team.
iii) Finally, environmental interventions were conducted in the schoolyards. Fixed (a balance circuit and panels with incentives to be physically active during recess) and mobile equipment (tyres of different colours and sizes) were put in the playgrounds to encourage children to be more active during playtime.

The standard physical education curriculum (one h/week of psychomotor activities to third year of pre-school children and two h/week of physical education to first grade of primary school children with physical activity levels at low-to-moderate intensity) continued to be taught in both the control and intervention schools because it is compulsory in Spain.

In the second year the CG became IG and the IG became the CG.

#### Organisation and functioning of the MOVI-KIDS program

The MOVI-KIDS program has been coordinated by two Physical Activity Sciences graduates, and implemented by monitors with technical qualifications in PA and sports, Physical Education teachers, or Physical Activity Sciences graduates. The monitors were trained over two days in order to standardise the performance of program activities.

#### Attitudes and adherence

To encourage adherence to MOVI-KIDS, children attending to a minimum of 80% of the trimester sessions were positively reinforced received small gifts depicting the logo of the program’s mascot.

#### Evaluation and follow-up

Besides a telephone number, an email address, and the blog (http://movi3kids.blogspot.com.es/), available for parents and teachers over the two years, two meetings were conducted with monitors; one at the baseline and the other one three months after the beginning of the program. Monthly contacts with monitors were held by phone and e-mail to get information on schoolchildren attendance to the program. And a quarterly visit to the centres was made to assess program performance and conduct satisfaction surveys among children.

#### Intervention with parents and teachers

Before the beginning of the program researchers, teachers, and parents were asked to decide the best way to implement modifications in the playground, and to discuss strategies for the family support of MOVI-KIDS. During the program, actions were taken to involve teachers and parents in the IG in promoting healthy lifestyles in children (please, see above).

### Study variables

Baseline and post-intervention outcome variables are going to be measured in both CG and IG three times, at the beginning and at the end of the first academic year, and at the end of the second academic year (in September 2013, June 2014, and June 2015). Table 1 provides an overview about all variables measured.

**Table 1:** **Study variables**

**Type of variable**	**Specific variables**
Primary endpoints	Anthropometry: weight, height, body mass index, waist circumference, triceps skinfold thickness, percentage of body fat and fat free mass by bioelectrical impedance analysis
	Blood pressure
Secondary endpoints	Physical fitness: cardiorespiratory fitness, muscle strength, speed/agility, flexibility
	Physical activity: parent’s report by the Netherlands Physical Activity Questionnaire (NPAQ), accelerometry
	Carotid intima-media thickness
Other endpoints	Densitometry
	Energy expenditure in playground games included in MOVI-KIDS and mean energy expenditure of a standard session of the program
	Economic evaluation: cost-effectiveness analysis
	Children’s perceptions about the school environment, and how this determined the physical activity development: focus groups, participant observation, and weekly diaries
Potential confounding factors	Age
	Sex
	Birth weight
	Breastfeeding: breastfeeding, formula-feeding, or both (mixed feeding)
	Food consumption: parents report by Children’s Eating Habits Questionnaire (CEHQ)
	Familial socio-economic status: education, occupation, and socioeconomic status index
	Area (urban or rural)
	Origin (native or foreign)

Anthropometrical variables (weight, height, BMI, waist circumference, triceps skinfold thickness, percentage of body fat, fat-free mass and blood pressure) were collected on the standardised conditions as were extensively described in the MOVI-2 protocol [26].

The measures were made in the school by trained investigators to minimise inter-observer variability.

#### Primary outcome measures

Anthropometry and body composition. *Weight* was measured twice (Seca® 861 scales) with the child barefoot and in light clothing. *Height* was also measured twice, using a wall stadiometer (Seca® 222), with the child barefoot and upright and with the sagittal midline touching the back board. BMI was calculated as weight in kg divided by the square of the height in m. *Waist circumference* was measured three times at the midpoint between the last rib and the iliac crest at the end of a normal expiration and using a flexible tape. *Triceps skinfold thickness* was measured three times at the triceps using a Holtain Ltd. caliper (0.2 mm accuracy and consistent 10 g/mm2 pressure between valves). The *percentage of body fat* and the *fat-free mass* were estimated with a four-electrode Tanita® Segmental-418 bioimpedance analysis system (Tanita Corp. Tokyo, Japan). Two readings were obtained in the morning, under controlled temperature and humidity conditions, with the child being shoeless and fasting, and after urination and a 15-minute rest. The mean of all the anthropometric measurements was calculated and considered for the analysis.

#### Blood pressure

Systolic and diastolic blood pressure were measured twice after a five-minute resting period, with a five-minute interval between measurements, using an OMRON-M5-I automatic tensiometer (Omron Healthcare UK Ltd.). Two readings were obtained and the mean of them was considered for the analysis.

#### Secondary outcome measures

Physical fitness. After four minutes of guided warming up, the main components of fitness [29] were measured as follows:

- Cardiorespiratory fitness [30], using the Course Navette test (20-minute shuttle run test), which was validated to measure the maximal aerobic capacity in children at least five years old, and performed using the Leger protocol.
- Muscle strength, using the standing long jump that measured explosive lower body strength.
- Speed-agility, using the 4×10 shuttle run test. Two attempts were made with an interval of five minutes, and only the best mark was used for analysis.
- Flexibility, using the sit-and-reach test that measured the maximum distance the participants could reach with their fingertips by flexing the trunk without flexing the knees. Participants could make three attempts and only the best mark was used for the analyses.

#### Physical activity

This was evaluated using the Spanish version of the Netherlands Physical Activity Questionnaire (NPAQ) for parents [31], who provided information about their children’s daily activity preferences during the six previous months. In addition, accelerometers GT3X were used for seven days consecutively (including nights) to measure the PA in children from six schools in the IG, and another four in the CG in the first academic year. Accelerometers were programed in epochs of one second and data were analysed using KineSoft software, version 3.3.2.0.

#### Carotid intima-media thickness

A carotid ultrasound measurement was made in a subgroup of 200 students (100 from IG and 100 from CG) to evaluate the stiffness index with a Sonosite Micromax Ultrasound, with a lineal translator multi-frequency high resolution 5–10 MHZ with Sonocal software, which makes automatic measures of the medium and minimum intima-media thickness.

#### Other study end points

Densitometry (DXA). A densitometry was made in the same subsample of 200 students to more accurately estimate fat mass, lean body mass, and bone mineral density.

#### Energy expenditure in playground games included in MOVI-KIDS

This was estimated in 40 students of the IG using the oxygen consumption measured by a portable gas analyser (Cosmed K4b2, Roma, Italia) [32]. Moreover, the mean energy expenditure of a standard session of MOVI-KIDS was estimated using the same procedures.

#### Economic assessment of MOVI-KIDS

A cost-effectiveness analysis following a societal perspective was used [33]. The difference between MOVI-KIDS cost and the children’s health care cost if they did not participate in MOVI-KIDS was calculated. The effectiveness was calculated as the differences in health variables (fat mass percentage, fitness) between the control and intervention groups.

#### Barriers and facilitators

The qualitative methodology was used to know the children’s perceptions about the school environment, and how this determined the PA development.

Weekly diaries of family activities, participant observation during the break time, and focus groups with parents and teachers were used. Using saturation data techniques [34] we performed six to eight focus groups with parents or caregivers and four to six focus groups with teachers [35], all selected by deliberate sampling techniques, and 10 family diaries.

Two expert researchers in qualitative methodology (a moderator and an observer) carried out the focus groups. The focus groups were recorded in audio and video, with the authorisation of the participants. Subsequently, the recordings were transcribed, and the texts were organised.

The participant observation was developed by a researcher, who collected the data in a field diary. The family diaries were self-reported following indications of the researchers. Data were analysed using a phenomenology perspective in order to deeply understand the motives and beliefs that influence the behaviours [36] . The data were processed and analysed using the Atlas-Ti 5.0 program. Two independent researchers carried out the analyses.

#### Confounding variables

Age, sex, birth weight, breastfeeding, food consumption, socio-economic status, living area (urban or rural), and origin (native or foreign) were considered potential confounding variables.

#### Breastfeeding

Mothers were first asked what type of feeding, breastfeeding, formula-feeding, or both (mixed feeding) had been chosen during their child nurturing. They were asked up to what age the child was exclusively breastfed and when the child completely stopped breastfeeding. In exclusive breastfeeding, the infant received exclusively breast milk without any supplementary feeding.

#### Food consumption

It was estimated using the Spanish version of the Children’s Eating Habits Questionnaire (CEHQ), validated for children from 2-to-9 years old. This version should be completed by parents [37].

#### Familiar socio-economic status (SES)

Data regarding familiar SES were gathered by using self-reported occupation and education questions completed by either the father or the mother. The paternal and maternal education was classified separately as primary education (functionally illiterate, without any studies or those not completed primary education), middle education (primary education, high school/secondary education or ‘Bachillerato’) and university education (university degree or PhD). Parental occupation was classified into five categories as follows: 1. Supervisor/manager or freelance with 10 employees or more; 2. Supervisor/manager or freelance with less than 10 employees, 3. Freelance with no staff; 4. Non-qualified staff and unskilled worker; and 5. Household chores, unemployed, and others. An index of SES was calculated using the items regarding parent’s education and occupation. This index distinguish, according to the scale proposed by the Spanish Society of Epidemiology, five categories of familiar SES: Lower, Upper lower, Lower middle, Upper middle, and Upper [38].

### Statistical aspects

The sample size was estimated to be able to show differences between the CG and the IG of 2% (alpha error of 0.05 and statistical power of 0.80) in mean body fat at the end of the first year. The estimated sample size was 140 children per group; this figure was multiplied by an inflation factor for cluster-randomised trials [39] which was estimated at 1.1264 using measurements from previous studies in BMI [20]. In order to examine subgroups differences (i.e.: sex, age, or SES) under the same conditions and estimating a 15% dropout rate, the minimal sample size was estimated in 1,600 children (800 per group).

The statistical analysis was carried out in two phases. In the first, we verified that the randomization had been effective in creating two comparable groups, examining the differences between the CG and IG in the mean body fat and cardiorespiratory fitness. After that, we identified outliers and extreme values, and their trustworthiness. Finally, we checked the adjustment of the variables to the normal distribution by the Kolmogorov Smirnov test and graphical procedures (normal probability plot).

In the second phase, we are going to assess the change between the intermediate and final evaluation variables at the first and second years of the study. Mixed regression models [40] will be estimated using each outcome variable as a dependent variable at the end of the first and the second years of the study. The different models will be adjusted for baseline values, age, and school (cluster), and the interventions will be treated as fixed affects (1 = IG; 0 = CG).

The results will be expressed as absolute differences in change on variables between the baseline and the final measurements (IC of 95%). When the dependent variable to be the prevalence of overweight or obesity, the odds ratio (OR) will be calculated together with a 95% IC.

Because of the different patterns of weight and height, triceps skinfold thickness, and body fat, the models will be separately implemented for boys and girls. A separate analysis will be done by sex, SES, origin (native or foreign), area (urban or rural), and public or private school.

Analyses will be carried out taken into account the CONSORT norms for the publication of the cluster design studies [41] with an intention to treat perspective, with children maintained in the IG or CG to which they were originally assigned, regardless of the number of sessions they attended.

Results will be considered statistically significant at p < 0.05, and the analysis will be performed using the 9.1 version of the SAS statistical package [42]. To the mixed generalised lineal models, PROC GENMOD will be used for dichotomous variables and PROC MIXED for continuous variables.

## Discussion

The aim of this paper was to describe the rationale and methods of a cluster randomized cross-over trial aimed to test the effectiveness of a PA program in children on preventing obesity during the adiposity rebound period, conducted in the school environment. The MOVI-KIDS program was conducted in the school environment, and has several strong points. First, it is a program that takes into account the parents’ and teachers’ opinions and preferences without overburdening them. Second, it does not require any changes in the curriculum and includes innovative aspects in the physical structure of the schoolyard, because some studies suggest that painting the schoolyard with attractive colors and providing it with permanent or fixed equipment increases the time that children spend in moderate or vigorous PA [43,44]. Third, this program was designed according to the socio-ecological model paradigm, thus the inclusion of the main school’s stakeholders (teachers, parents, school board) during the whole process of the program implementation might help circumvent the influence of some barriers for the PA that the qualitative research approach could identified. Finally, it seems judicious to think that the interventions aimed to improve adiposity levels in Spanish children should be mainly focused on the promotion of PA because of: i) the low physical fitness levels of the students; ii) it has been reported data suggesting that an excess of energy intake might not be necessarily the main driver of the obesity epidemic in Europe; iii) a diet intervention could increase the risk of thinness in children, and iv) an intervention based on playground games could improve other aspects such as quality of life, academic achievement, and quality and time of sleep. Thus, it seems necessary to assess the effectiveness of the interventions to prevent the overweight, in order to prevent the cardiovascular morbidity and mortality of the future. Moreover, the timing when the adiposity rebound occurs might be, along with pregnancy and breastfeeding, one of the most important periods for preventing children and adolescent obesity, and the school is the most suitable place to develop these interventions.

## Abbreviations

PA: Physical activity
CG: Control group
IG: Intervention group
SES: Parental socio-economic status
